# Pathological and Therapeutical Observations on Hæmorrhoids

**Published:** 1818-10-01

**Authors:** 


### Medico-Chirurgical Journal;

OR,

### Quarterly Register

OF

### MEDICAL AND SURGICAL SCIENCE.

Quarterly Series.] OCTOBER 1, 1818. [Vol. I.

### First Department.

### PRACTICAL ESSAYS AND COMMUNICATIONS,

*Original or Translated.*

### I.

### *Pathological and Therapeutical Observations on Hæmorrhoids.*

(Analysed and Translated from the French of Montegre.)

"The subject of the present Article is characterized by laborious and patient research, and exhibits all the systematic arrangement and analytical transparency, which are peculiar to the scientific literature of that country." *Sir T. C. Morgan, in the Edinburgh Journal, No. LIII.*

IN the highly respected cotemporary Journal above mentioned, is given a meagre and imperfect account of an extremely interesting article in the 20th Vol. of the great French work now publishing by some of the most eminent physicians and surgeons of France. We shall here present the English reader with a clear and well defined picture of Dr. Montegre's monograph, convinced that the subject is both ill understood and too much neglected by British physicians. Having been *hæmorrhoidinarians* ourselves for more than twenty-five years, and having paid unusual attention to this complaint, both in our own persons, and in our patients, we may be allowed to be, in some degree, judges of the doctrines and practices brought forward by the continental physicians; consequently, this article is to be regarded not as a tame translation, but as an *eclectic* analysis, occasionally interwoven with the personal observations of the Translator.

Sect. I. Description and Definition.

The well known Greek definition of hæmorrhoids [sanguifluxus] conveys a most imperfect idea of the disease, since a *discharge of blood,* though a frequent, is by no means a constant symptom. The same may be said of the anal tumours. From the effect of causes which it is often difficult to recognize, there occurs, at certain periods, a sanguineous fluxion or determination (fluxion sanguine; mouvement fluxionnaire) towards the extremity of the rectum, evinced at first by merely a sense of *tension or weight* there, with scarcely any pain, and gradually going off in three or four days. This determination is renewed at longer or shorter intervals; frequently, but not uniformly, accompanied by a discharge of florid blood on going to stool, exhaled, as it were, from the mucous membrane of the intestine, and usually without any visible erosion of structure. After a time, hæmorrhoidal tumours in general appear, merely as a *consequence,* but not as an essential part of the affection. In fact, the sense of *tension and weight* is the only *pathognomonic* mark of the disease; all the others are accessory, or contingent.

In well marked cases of hæmorrhoids, there are certain general or constitutional symptoms, which have been clearly, though concisely, summed up by the venerable Pinel. "Slight horripilation of the back and loins; occasional numbness of the lower extremities; hardness and concentration of the pulse; pallor of the face; dullness of the eyes, with a dark circle round them; dryness of the mouth; scantiness of the urine; gastric debility; intestinal flatulence; frequent inclination to stool and urine; sense of pressure about the anus and perinæum, with an occasional discharge of mucus." *Nosograph. Philosoph.*

It is hardly necessary to remark, that this train of symptoms, which evinces a great fluxionary movement in the system, is sometimes so slight as to evade notice, and is only recognized by the sanguineous discharge.

From this view of the subject, a considerable analogy between the hæmorrhoidal and menstrual flux will at once be perceptible, though the *periodicity* of the latter is much more regular than that of the former. We shall not, however, dwell on this analogy; but we may remark that the connexion between the hæmorrhoidal and *portal* circulation,* in both sexes, points not only to the *causes*, but to the salutary *effects* of the hæmorrhoidal discharge, in various derangements of the organic balance. When within certain bounds, therefore, it can hardly be called a disease; since without impeding the exercise of any function, or causing any material inconvenience or pain, it tends, like the menstrual flux, towards the conservation of health.

---

Sect. II. Accessory Phænomena or Complications.

These are commonly hæmorrhages; tubercles and tumours; inflammation; leucorrhœal discharges; *or consecutively*, fissures; excrescences; pains or strictures of the rectum; ulcerations; abscesses; fistulæ; indurations or even scirrhi of the gut; prolapsus ani; inflammation or irritation of the bladder or neighbouring parts. Of these contingent phenomena, we shall only notice one or two.

*Hæmorrhage.* This is among the first contingencies that supervene on the hæmorrhoidal movement, and is that which has improperly given a name to the affection. The most usual species of hæmorrhage is by a kind of *exudation* or exhalation from the extremities of the capillary vessels of the mucous membrane, in the same manner as from the uterine vessels at the catamenial periods. The blood in these cases is vermillion-coloured, accompanying, but not mixed with the fæcal matters. Sometimes the blood issues by a fine and continued jet, during the straining at stool; and yet, when we examine the parts with glasses, no solution of continuity is to be found, the blood evidently having issued from a dilated pore. Professor Richerand records a remarkable example. "A merchant arrived at his 90th year in perfect health. This long immunity from disease he attributed to an hæmorrhoidal flux which had been regularly established more than fifty years, and so considerable in quantity, that the blood spouted to a certain distance from the anus, as from a vein opened by a lancet." *Nos. Chir.*

*Active Hæmorrhage.* This is the result of a vital movement, and is always salutary when in moderation. It may, however, prove dangerous when excessive, and lead to *passive* hæmorrhage, which is of a much worse character. The immoderate or inconsiderate use of venesection from

---

* "Vena Portæ, Porta Malorum." *Stahl.*

the system at large, or application of leeches to the anus itself, frequently determines this excess. The quantity of the hæmorrhoidal discharge varies much, as there are innumerable examples of people losing from a few drops to one or even two pounds of blood *daily*, and that for a long time, without any injurious consequences. Montanus knew an hæmorrhoidinarian, who for forty-five days in succession, discharged more than two pints of blood *per anum*, daily, and yet he perfectly recovered. Panorola relates the case of a noble Spaniard, who, during four years, passed daily a pint of blood by stool, and yet enjoyed the most perfect health. Boulli notices the case of a tailor, who passed torrents of blood from the hæmorrhoidal vessels, sometimes to the amount of ten pints; yet was he vigorous, and of a sprightly jovial character. Hoffman relates the case of a widow lady, 50 years of age, gross, and a high liver, who after being harassed with a variety of anomalous symptoms, and particularlylassitudes, languors and faintings, was seized with the hæmorrhoidal flux, and in twenty-four hours lost more than *two gallons and a half* of blood! The symptoms of debility and oppression abovementioned were quickly dissipated, and she gradually recovered health and strength. These examples are sufficient to assure us, that the hæmorrhoidal discharge may be often enormous, without being necessarily fatal or even dangerous.

For the nature of the hæmorrhoidal tumours themselves, we refer to the short extract from the work of Larroque, inserted in this number of the Journal.

*Mucous or Serous Hæmorrhoids.* These occasionally succeed the sanguineous forms of the disease, especially where much inflammation has attended. They bear a very exact similitude to the leucorrhœal discharges from the uterine vessels, and depend on the same general and local causes. It is something remarkable, that they seldom occur where the sanguineous discharge from the hæmorrhoidal vessels is copious; a circumstance in which they correspond with the whites, which are rarely seen in females who have copious catamenia.

---

### Sect. III. Classifications and Distinctions.

Dr. Montegre divides hæmorrhoids into two orders: the regular or periodical; the anomalous or irregular. The

*first* are generally constitutional, and not to be cured but at some risk. Such, for instance, as alternate with or succeed the catamenial discharge. The *anomalous* order are usually dependent on accidental circumstances, and the removal of their *causes* is the proper method of cure. These two orders are divided by our author into eight species, comprehending numerous varieties. We shall briefly glance at the Species. 1. The dry hæmorrhoids (cæcæ). These offer the genuine hæmorrhoidal movement or determination to the rectum before alluded to, and have no varieties. 2d Species. The H. fluentes, with two varieties; the sanguineous and mucous. 3d Species. H. tumentes, with two varieties; the varicosæ and the mariscæ. 4th Species. H. dolentes, with three varieties; the inflammatoriæ, nervosæ, and H. cum fissuris. 5th Species. H. cum contractione ani. 6th Species. H. cum ulceratione. 7th Species. H. cum procidentia ani. 8th Species. H. cum irritatione vesicæ urinariæ.

We do not mean to follow our author through these orders, species, and varieties; but we think the classification a useful and natural one for any author or lecturer who treats fully and distinctly on the subject. No one can deny that these *modifications* of the disease actually present themselves in practice, and moreover that they require a corresponding *modification* of treatment and attention. They are not therefore *useless* distinctions, as the indolent would fain represent them.

---

### Sect. IV. Etiology.

1. Hereditary predisposition. 2. Climate. 3. Age. 4. Sex. 5. Habits of life. 6. Season and temperature. 8. Food. 9. Constipation. 10. The labours of the cabinet. 11. Depressing passions. 12. Certain diseases. 13. Pregnancy. 14. Tight clothes round the abdomen. 15. The abuse of drastic purgatives. 16. Too frequent use of glysters. 17. Venery. 18. External irritation. 19. The use of hollow night stools.

On each of these points Dr. Montegre descants with great acuteness and knowledge of the subject, and we regret that our limits forbid our following him.

We have so invariably found strong mental emotions of a *melancholy nature* induce the hæmorrhoidal movement in our own persons, and have so often observed the same in others, that we can confirm the statements of our author on this head.

"Anger, fear, ennui, disquiet, and habitual melancholy, says Dr. Montegre, exert a remarkable influence on the *cœliac plexus*, situated in the abdomen, and in immediate sympathetic communication with the liver, biliary ducts, and the whole system of vessels that return the blood from the rectum. This impression is most felt at the epigastrium by a sense of uneasiness, load, and kind of constriction there, producing derangement of the digestion, and biliary secretion, gastric irritability, &c. The effect of these melancholy emotions is to unhinge the balance of the circulation, and give origin to internal concentrations of blood. The cutaneous vessels become almost exsanguious, and it is under such circumstances that we see rupture of the parietes of the heart, or fatal congestions in some of the other viscera. Here an explosion of the hæmorrhoidal flux may often ward off the impending danger."

Under the head of *medicinal causes* of hæmorrhoids, Dr. Montegre enumerates the frequent use of aloes, rhubarb, neutral salts, [on the authority of Hildebrandt], emmenagogues, certain mineral waters, glysters, &c.

*Miscellaneous causes.* Long and fatiguing marches on foot; *violent* horse exercise [gentle horse exercise is one of the most powerful anti-hæmorrhoidal measures] sudden impression of heat or cold on the region of the fundament; the too frequent application of leeches to the anus; the inordinate use of foot baths; [and we may add of biddies]; the use of hollow seats [usage d'un siege percé] and lastly, according to the opinion of De Haen, the effluviæ from privies while seated at stool.

---

### Sect. V. Prognosis.

In this section Dr. Montegre enters deeply into the argument that, speaking generally, hæmorrhoids are salutary efforts of the constitution, although they may often, like many other sanative processes, prove injurious, or even fatal. Attentive observation will shew that, in the present state of society, almost every individual of the human race has some one organ or part of the body more weak, more irritable, or more predisposed to disease than the rest; and that this part is usually an important viscus of vital function. This consideration will induce the pathologist to acknowledge, that the hæmorrhoidal movement and discharge are, upon the whole, productive of salutary effects, as determining this irritation or local plethora to a part where it may be expended with safety, though with inconvenience or pain.

Let us apply this reasoning. M. Bayle and others have shewn, that pulmonary consumption carries off a fifth part of the human species. Without entering into a disquisition on the nature of phthisis, it will be very generally allowed that it consists essentially and originally in a *sanguineous determination* to the respiratory apparatus, however slow or insidious its progress. Now, granting that the causes, whatever they may be, which induce such a wide wasting disease, are operating with more or less intensity on all classes, we have strong reasons for believing that the hæmorrhoidal movement preserves, in a considerable number of cases, the lungs from the disorganizing irritation of phthisis. This reasoning is strongly supported by the well known connection which subsists between pulmonary consumption and fistula in ano, the latter almost constantly arresting the progress of the former; and on the contrary, very often giving origin to the pulmonic affection when suddenly cured in unsound constitutions. If, then, *fistula in ano*, which acts like a perpetual hæmorrhoidal drain, can arrest the progress of phthisis, after it has actually been developed, as every observant practitioner must have repeatedly seen, how much reason have we to expect that an establishment of the hæmorrhoidal flux, *anterior* to phthisis, may in many cases, avert it entirely?

The following case from Larroque may come in here as illustrative of the subject.

"A lady, previous to puberty, had all the symptoms of pulmonary consumption; but as soon as the menses were established, these symptoms disappeared, although several distinguished physicians had pronounced the case incurable. From this time till the cessation of the menstrual discharge, at the age of 45, no complaint of the chest was manifest; but at the turn of life the symptoms of phthisis were again renewed. Fortunately at this epoch the hæmorrhoidal flux appeared, and the thoracic affection instantly gave way. Between the age of 60 and 70, the hæmorrhoidal discharge ceased, and again returned the cough and expectoration of which she died."

The following case, communicated to Dr. Montegre by Dr. Bodson, exhibits another example of the salutary effects of the hæmorrhoidal flux, where there is reason to fear that an important organ is on the eve of structural lesion.

A man, 25 years of age, married two years, tall and thin, became affected with constant and severe pain between the shoulders, accompanied by cough and copious expectoration, emaciation, and progressively increasing debility. Notwithstanding various means, these symptoms got worse and worse, and the young man was considered to be in a confirmed consumption. The attending physician happening to recollect that the father of the patient had been hæmorrhoidinary, conceived that the establishment of such an affection might be serviceable to the son, and consequently applied six leeches to the fundament. The effect was so rapid and decisive, that it appeared as though the pulmonary disease was destroyed by a single blow. The hæmorrhoidal movement became irregularly established, he recovered flesh and strength, and continued in good health."

Indeed, since the days of Hippocrates, hæmorrhoids have been considered by accurate observers to have an ani-phthisical tendency. The Father of physic distinctly says,—"*Qui sanguinem per ora venarum quæ sunt in ano, perfundere solent, ii neque lateris dolore; neque pulmonis, inflammatione corripiuntur.*" *De Humor.*

From all these considerations, then, would it not be a useful indication to endeavour to establish the hæmorrhoidal discharge by aloetics, leeches, &c. in those who are predisposed to phthisical disorders in this country?

---

### Sect. VI. Retention and Suppression of Hæmorrhoids.

The *causes* of these accidents may be divided into preparatory and efficient. Among the first order we may reckon the nervous temperament, great sensibility, a predisposition to morbid action in some internal organ or part, as the bladder, kidnies, liver, stomach, lungs, heart, or great vessels.

The *efficient* causes of retension are very numerous. The principal are, high inflammatory action in the system, excessive hæmorrhages, violent mental commotions, as terror, anger, &c.; the application of inordinate heat or cold to the parts; wet and cold to the feet; baths too hot or too cold, taken during the paroxysm; aliments very high seasoned or stimulating; inordinate evacuations, whether from the vascular, lymphatic, or glandular systems, as profuse venesection, great sweats, salivation, hypercatharsis, &c.; the application of irritating or astringent substances to the hæmorrhoidal tumours.

*Consequences of Retention and Suppression.* It must be borne in mind, that the most sudden and abrupt suppression of a long established hæmorrhoidal flux, whether by accident or by topical remedies, will, in very many cases, be followed by no inconvenience, and the patient may enjoy many years of perfect health, and immunity from the original affection. In illustration of this position we could relate numerous facts, but shall confine ourselves to one or two instances.

*Case* 1. "A gentleman, 34 years of age, very robust, of a bilio-sanguine temperament, born of hæmorrhoidinary parents, and long afflicted with most violent paroxysms of the complaint, sometimes accompanied with sanguineous discharge, sometimes not; observed that the intervals of health were always long, in proportion to the force and duration of the preceding attack. After an unusually severe hæmorrhoidal paroxysm, which was procrastinated six months, and attended with great pain, he was suddenly cured by a topical application. He has now continued five years free from hæmorrhoids and all other complaints, although he leads the same kind of sedentary life as before, and often keeps late hours."

*Case* 2. "My father, then 22 years of age, happening to be travelling in Italy, was afflicted several months with the most excruciatingly painful hæmorrhoids, accompanied by a sanguineous discharge and external tumours. He was cured in one night by an empirical application at Venice, and never afterwards experienced any hæmorrhoidal affection. He died of apoplexy at the age of 78."

The prognosis, then, in cases of sudden suppression of the hæmorrhoidal movement, must be founded on the nature and importance of the effects which follow. The suppression will be more dangerous in proportion as the individual is predisposed to any visceral affection, as phthisis, cardiac disease, aneurism of any of the large vessels, &c. Advanced age, and the turn of life in females are unfavourable epochs for such accidents.

But although immunity from disease frequently follows a suppression of the hæmorrhoids, we are by no means to calculate on such good fortune as even generally to be met with. We shall here then present a rapid sketch of the various phænomena which attentive observation has ascertained, as very frequently resulting from the suppression or retention under consideration.

1. *Fever* has, in many instances, been kindled up by the suppression of the hæmorrhoidal flux. Ludolph relates a remarkable instance:—A man of letters, 40 years of age, thin, yet plethoric, of sedentary habits, had frequently experienced the hæmorrhoidal discharge with advantage to his general health. But this discharge having become excessive, his physician suddenly suppressed it; the consequence of which was pains and sense of anguish at the præcordia, acute fever, violent delirium, and death in a few days! Stahl offers us nearly a similar example.

2. *Phlegmasiæ.* The brain or its meninges, the lungs or their coverings, the heart, the stomach, the liver, and peritoneum, are often affected with inflammation from suppressed hæmorrhoids. But chronic engorgements, with gradual induration of these viscera, are the more usual results.

3. *Hæmorrhages.* Almost every part of the body may become the seat of hæmorrhage, *vicarious* of the hæmorrhoidal flux when suppressed; but more especially the uterus, the bladder, the stomach, the liver, and the lungs.

4. *The Neuroses.* Esquirol asserts, that melancholy and insanity frequently result from the suppression of hæmorrhoidal evacuations; Poissonier, Andry, &c. saw tetanus result from the same; Heister, hypochondriacism; and, according to Dion Cassius, the Emperor Trajan experienced an attack of apoplexy, followed by hemiplegia, in consequence of a sudden suppression of the hæmorrhoidal flux, to which he had been long subject. He soon afterwards became dropsical, and died.

---

### Sect. VII. Treatment.

1. *The Hæmorrhage.* This accident demands our attention, since its *excess* may prove dangerous or even fatal, and yet its continuance may be necessary to the constitution. As long as the flow of blood returns periodically, with considerable intervals, and without apparently injuring the health, we should be cautious how we arrest the discharge; but when it produces sudden debility, paleness, or spasms, we should endeavour to moderate the flow. It is certain, however, that the most excessive hæmorrhage in this way is rarely fatal in itself, though it may prove so consecutively. It has been justly observed, too, that the greatest hæmorrhage is often less dangerous than the means which we take to arrest it.

These means should first be *general* rather than local. The patient should be laid on a cool bed in the middle of an airy room, with few or no bed clothes, and kept in the most perfect quietude in the horizontal position, his feet a little raised: if he can lie on his face, so much the better. These precautions, with cold acidulated drink, will commonly *moderate* the discharge, which is better than suddenly checking it. If these means are unsuccessful, we must endeavour to induce a determination to some other quarter, by venesection, by cupping glasses to the shoulders, by ligatures to the upper extremities, as practised by Galen, and too much neglected by the moderns; by long continued frictions, with flesh brushes, to the upper parts of the body, especially in the latter or passive stage of the hæmorrhage. On the same principle of counter-derivation, we apply sinapisms to the inside of each arm. When these means fail, we must have recourse to *local* applications, such as cold, astringents, or plugging the rectum.

The ancients were in the habit of applying the actual cautery, and Montegre recommends the same as a last resource.

2. *The Tumours.* The great art in the treatment of hæmorrhoidal tumours is *compression.* We can assert, from personal experience, that however large these bodies may project at stool, or at other times, if the patient lies down on his back, and makes gradual but constant pressure with his fingers, they will be almost always got within the sphincter ani, and by a T bandage well secured, with a small pad on the anus, they may be prevented from prolapsing.

The extirpation of hæmorrhoidal tumours by the knife or ligature, need not be touched upon in this place.

3. *Hæmorrhoidal Inflammation.* If no sinister accident take place, the hæmorrhoidal inflammation commonly runs a course of six or eight days, with considerable intensity, when the tumours begin to subside, especially if a discharge of blood occurs, re-enter the anal sphincter, and the pains vanish entirely.

To moderate the inflammation of hæmorrhoidal tumours, the application of leeches to the parts is a very general practice. Dr. Montegre reprobates this remedy, and certainly we have not experienced that advantage from this mode of local bleeding that we expected. Dr. M. asserts, that instead of unloading the gorged vessels, "the effect is quite contrary—the determination of blood to the part is augmented considerably, together with all the consequences of this determination." *L'effet en est ordinairement tout contraire; la fluxion est presque toujours considerablément augmenté, &c.* To the *loins* he advises the leeches to be applied, where they will have the effect of degorging locally, without producing irritation by their bites.

The application of cupping glasses, with or without scarification to the shoulders, or hypochondria, are recommended as very useful derivatives in excessive hæmorrhoidal discharges. Warm fomentations or half-baths, though popular, are in general hurtful remedies. M. Recamier has seen the tumours become gangrenous from their use.

We have, ourselves, however, seen very good effects from emollient cataplasms in painful hæmorrhoidal swellings that could not be pressed within the sphincter ani.

While these means are used, the patient should take mild cooling laxatives internally, as supertartrite of potash, tamarinds, manna, sulphur, &c. with cool diluting drinks.

4. *Hæmorrhoidal Pains.* Dr. Montegre calls the attention of the Faculty to the use of injections of cold water, or water with the chill off it, together with semicupia of the same, in those hæmorrhoidal pains attending the fæcal evacuation, accompanied or not with inflammation, which are so distressing to many people. He considers this remedy as, by far, superior to every other, while it is totally devoid of danger or inconvenience. We think this hint worth attending to.

5. *Equitation.* Although violent horse exercise will often occasion hæmorrhoids in those who are not accustomed to ride, yet there is not a more powerful remedial measure, when moderately used, than this. Indeed, no man need suffer from the piles who can keep a horse.

7. *Hæmorrhoidal Colic.* As this is occasioned by a determination of blood to the mesenteric vessels, there is no more speedy means of relief than by establishing the hæmorrhoidal discharge, by the application of leeches to the anus, warm emollient glysters, and purgatives.

8. *Constipation.* This is not only a very frequent cause of hæmorrhoids, but it is an accompaniment which renders the complaint one of the most painful afflictions of human nature!

*General Remedies.* Every thing which augments the cutaneous transpiration, and increases the activity of the absorbents of the intestinal canal; or, on the other hand, diminishes the secretory office of the mucous membrane of the intestines, tends to constipation. Hæmorrhoidinarians, therefore, ought to avoid all violent and unaccustomed exercises which cause much perspiration, as also hot and vapour baths. The diet must, of course, vary according to the idiosyncracy of the patient.

Of laxative medicines, the best are the tartrite of potash with sulphur and supertartrite of potash in molasses, as an electuary at night. But *injections of cold water* are, of all other remedies, by far the most efficacious. These injections should be small in quantity, not more than sufficient to fill the rectum.

In respect to the constitutional treatment of the hæmorrhoidal movement itself, it is needless to observe, that as our author considers this movement a salutary one, he rarely advises any means of counteracting it. On the contrary, where phthisis, apoplexy, or any organic disease of an internal viscus threatens the patient, Dr. M. thinks that a hæmorrhoidal discharge ought to be induced, or renewed, if suppressed.

We shall conclude this paper by an abstract of Dr. Montegre's

### GENERAL RULES OF CONDUCT FOR THE HÆMORRHOIDINARIAN.

A. A temperate climate agrees best with the hæmorrhoidary constitution. Too much heat stimulates the biliary system, weakens the digestive organs, and tends to constipation, by rendering the perspiration inordinate. A very cold climate, especially if moist, is also unfavourable to this complaint. Apartments heated and close, and transitions from thence to the cold air, ought to be particularly avoided.

B. The tepid, but not the hot bath, ought to form a part of hæmorrhoidinary regimen. In the intervals of the attack, the *cold* bath is extremely serviceable.

C. The local application of cold water to the fundament, every day, by means of a sponge, especially after going to stool, and when the tumours come out during the alvine evacuations, ought never to be neglected by the hæmorrhoidinarian.

D. The *clothing* should be such as is suitable in rheumatic complaints. Flannels next the skin, so as to favour the insensible perspiration, and defend the patient from abrupt atmospherical transitions, are necessary articles of dress. The bedding should be neither too soft nor too warm; and the hæmorrhoidinarian should avoid sitting on a seat that is either very damp and cold, or heated by the rays of the sun.

E. The *food* should not partake much of the hot, spicy, or stimulating; and the drink should be as aqueous as possible.

F. *Exercise.* Of all exercise, that of riding on horseback is the best. The swing is also useful.

G. " *L'acte venerien* est evidemment utile aux hemorrhoidaires, à moins qu'il ne soit repété d'une maniere excessive, ce qui incommode tout le monde."

H. The moral affections have a great and predominant influence on the hæmorrhoidal state. They ought therefore, to be attended to as much as possible, and kept in due bounds, though this is seldom within our power.

---

We have thus condensed the more important features of this long and interesting article in the French work; and we trust that we have, in so doing, rendered a service to the British medical reader.

